# “Epidemiology and aetiology of influenza-like illness among households in metropolitan Vientiane, Lao PDR”: A prospective, community-based cohort study

**DOI:** 10.1371/journal.pone.0214207

**Published:** 2019-04-05

**Authors:** James W. Rudge, Nui Inthalaphone, Rebecca Pavlicek, Phimpha Paboriboune, Bruno Flaissier, Chou Monidarin, Nicolas Steenkeste, Viengmon Davong, Manivanh Vongsouvath, K. A. Bonath, Melinda Messaoudi, Mitra Saadatian-Elahi, Paul Newton, Hubert Endtz, David Dance, Glaucia Paranhos Baccala, Valentina Sanchez Picot

**Affiliations:** 1 Communicable Diseases Policy Research Group, Department of Global Health and Development, London School of Hygiene and Tropical Medicine, London, United Kingdom; Faculty of Public Health, Mahidol University, Bangkok, Thailand; 2 Center of Infectiology Christophe Mérieux of Laos, Vientiane, Laos; 3 Naval Medical Research Centre (NMRC-Asia), Singapore, Singapore; 4 Fondation Mérieux, Vientiane, Laos; 5 University of Health Sciences, Phnom Penh, Cambodia; 6 Fondation Mérieux, Lyon, France; 7 Mahidol Oxford Tropical Medicine Research Unit, Vientiane, Laos; 8 Hopital Edouard Herriot, Hospices Civils de Lyon, Lyon, France; Public Health England, UNITED KINGDOM

## Abstract

Respiratory diseases are a major contributor to morbidity and mortality in many tropical countries, including Lao PDR. However, little has been published regarding viral or bacterial pathogens that can contribute to influenza-like illness (ILI) in a community setting. We report on the results of a community-based surveillance that prospectively monitored the incidence of ILI and its causative pathogens in Vientiane capital in Lao PDR. A cohort of 995 households, including 4885 study participants, were followed-up between May 2015 and May 2016. Nasopharyngeal swabs, throat swabs, and sputum specimens were collected from ILI cases identified through active case-finding. Real-Time PCR was used to test nasopharyngeal swabs for 21 respiratory pathogens, while throat and sputum samples were subjected to bacterial culture. Generalized linear mixed models were used to assess potential risk factors for associations with ILI. In total, 548 episodes of ILI were reported among 476 (9.7%) of the study participants and 330 (33.2%) of the study households. The adjusted estimated incidence of ILI within the study area was 10.7 (95%CI: 9.4–11.9) episodes per 100 person-years. ILI was significantly associated with age group (p<0.001), sex (p<0.001), and number of bedrooms (p = 0.04) in multivariate analysis. In 548 nasopharyngeal swabs, the most commonly detected potential pathogens were *Streptococcus pneumoniae* (17.0%), *Staphylococcus aureus* (11.3%), influenza A (11.1%; mostly subtype H3N2), rhinovirus (7.5%), and influenza B (8.0%). Streptococci were isolated from 42 (8.6%) of 536 throat swabs, most (27) of which were Lancefield Group G. Co-infections were observed in 132 (24.1%) of the 548 ILI episodes. Our study generated valuable data on respiratory disease burden and patterns of etiologies associated with community-acquired acute respiratory illness Laos. Establishment of a surveillance strategy in Laos to monitor trends in the epidemiology and burden of acute respiratory infections is required to minimize their impact on human health.

## Background

Respiratory diseases are a major contributor to morbidity and mortality in many tropical countries, including Laos. Endemic, emerging, and re-emerging respiratory diseases may threaten both local and global populations, as has been highlighted with the emergence of Severe Acute Respiratory Syndrome (SARS) in 2003 [1], followed by outbreaks of the highly pathogenic avian influenza (HPAI) H5N1 [2]. Among the 5,942 million deaths in children under 5 in 2015, 15.5% were due to pneumonia [3]. Southeast Asia is thought to play a particularly important role in the global circulation of respiratory pathogens such as seasonal influenza A/H3N2 [4–6]. However, epidemiological surveillance of respiratory pathogens in Southeast Asia has been limited compared with the western hemisphere [7]. Furthermore, the socio-economic, demographic and environmental factors that influence disease burden and transmission dynamics can vary substantially both within and between hemispheres [7,8]. Thus, characterizing local epidemiology and burden of respiratory pathogens is crucial for appropriate public health decisions.

Despite improvements in surveillance capacity for respiratory disease and influenza virus in many low- and middle-income countries (LMICs), particularly in Southeast Asia, there remain many challenges and knowledge gaps regarding the etiology and incidence of these diseases and the risks of pandemic or zoonotic influenza emergence [5].

In Lao PDR, national programs for pandemic preparedness and laboratory-based influenza surveillance were established following the emergence of HPAI-H5N1 [5,9]. Since 2006, the National Center for Laboratory and Epidemiology of Lao PDR (NCLE) has developed laboratory, surveillance, and epidemiological capacity and was designated as the World Health Organization (WHO) National Influenza Center in 2010 [10]. In 2009–2010, identification of the viral etiologies in 292 patients hospitalized for acute lower respiratory illness (ALRI) showed these were mainly due to rhinoviruses (35%), and respiratory syncytial virus (RSV, 26%). Other etiologic agents were influenza (12%), parainfluenza viruses (9%), adenoviruses (6%), Human *Metapneumovirus* (HMPV),and corona viruses (4%), and Human *Bocavirus* (HboV) (3%) [11]. *Streptococcus pneumoniae* and *Hempplilus influenza* were the main bacterial pathogens causing ALRI in hospitalized children [12].

The National Immunization Program (NIP) in Laos has made considerable progress since 2009. The pentavalent vaccine including *Hemophilus influenzae* type B is included in the NIP since 2009 [13]. With the assistance of Gavi, the country introduced the conjugated pneumococcal vaccine PCV13 into its NIP in October 2013. The schedule was for PCV13 vaccination to be given to infants at 6, 10, 14 weeks. The results of two cross-sectional community carriage surveys pre- and two years post-PCV13 introduction showed a significant 23% reduction in carriage prevalence of PCV13-related strains in 12–23-month-old children [14]. The prevalence of carriage of PCV13 strains was also lower in 5-8-week-old infants, suggesting evidence of herd immunity in this age group [14]. Japanese encephalitis vaccine and inactivated polio vaccine were introduced in 2015; and a second dose of measles-rubella vaccine targeting children 12–18 months in 2017 [13]. Despite a clear increase in NIP vaccines, pockets of suboptimal vaccination delivery and utilization persist, mostly in ethnic minority groups and remote rural areas [13]. A seasonal influenza vaccination program with trivalent inactivated was launched in 2012 for high-risk adults including pregnant women [15].

Community-based surveillance studies can provide valuable data for the estimation of epidemiological parameters and circulatory patterns of pathogens. The Longitudinal surveillance study of respiratory pathogens in metropolitan Vientiane, Lao PDR (LaCoRIS) is a community-based study that monitors the etiology and burden of respiratory disease among residents of this region. The project was initiated in 2015 as an extension of surveillance activities initially set up by CoPanFlu project in 2009–2010 [16]. The project was conducted by Fondation Mérieux in partnership with the Center of Infectiology Christophe Mérieux of Laos (CICML), Lao-Oxford-Mahosot Hospital-Wellcome Trust Research Unit (LOMWRU), and the London School of Hygiene & Tropical Medicine (LSHTM). The primary objectives of LaCoRIS project were to measure the incidence rates of acute respiratory diseases and to identify their etiologic pathogens in metropolitan Vientiane.

## Methods

### Study design and study area

**Fig 1:**
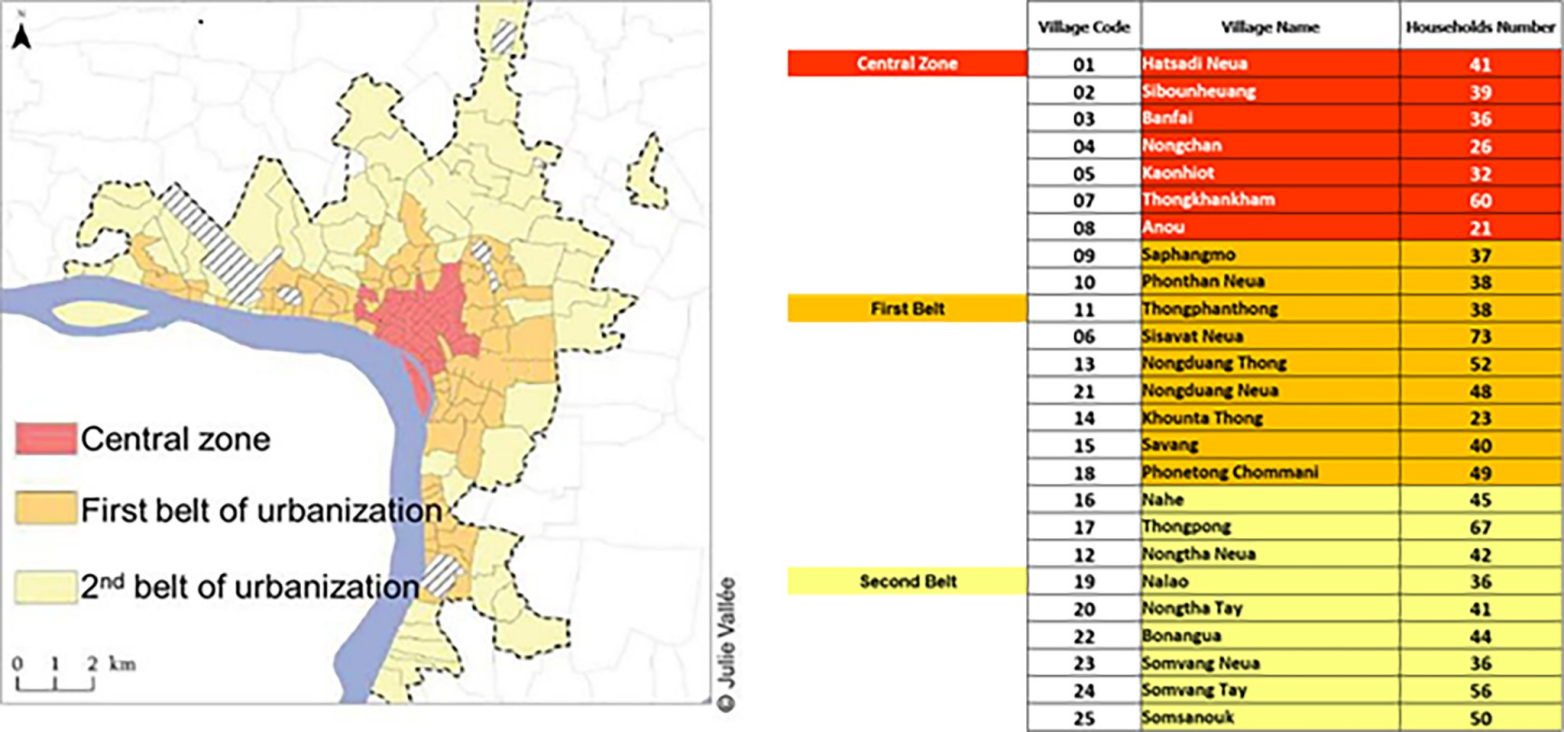
LaCoRIS catchment area.

This was a prospective, community-based, cohort study within the Vientiane metropolitan area in Lao PDR which has a total population of approximately 6.7 million, based on the 2016 census data [17]. The surveillance covered a total catchment area within the administrative boundaries of the Vientiane metropolitan area and included 4885 study participants in a total of 995 households in 25 villages (Fig 1). Households were selected from household registries and were enrolled into the cohort via door-to-door sampling. A household was defined as a group of individuals living on the same plot of land (one or more houses) or in the same building (apartment) and sharing their meals. All permanent residents of the household who met the inclusion criteria of > 6 months old and living in their respective village for at least 6 months were eligible to participate. Written consent was obtained from all participants before the start of the surveillance.

### Ethic approval

The study was approved by the Lao National Ethics Committee for Health Research in Lao PDR (060/NECHR; 01 January 2017), and the Research Ethics Committee of the London School of Hygiene and Tropical Medicine (reference 7653, 18 June 2014).

Upon enrollment, a household-level questionnaire was administered to collect data on socio-economic and potential exposure-related variables (including durable assets, water sources, sanitary systems, and animals owned). In addition, for each enrolled household member, individual-level data were collected on subject age, sex, pre-existing medical conditions and influenza vaccination history within the past 12 months.

### Surveillance and case investigation

**Fig 2:**
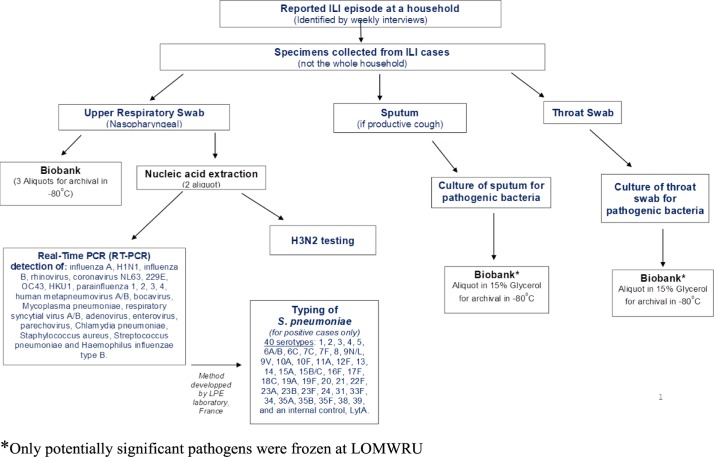
Flow chart of specimen collection and laboratory testing.

The study flow chart is shown in Fig 2. From May 2015 to May 2016, active case finding for influenza-like illness (ILI) was conducted among the cohort through daily phone calls by staff at CICML. The suspected ILI cases were first identified using the WHO case definition as either > 37.5°C axillary temperature or > 38°C tympanic temperature with an associated respiratory symptom, i.e. cough, sore throat, or shortness of breath with symptoms starting within 10 days [18]. As soon as a case fulfilled the ILI-WHO criteria, a free of charge visit was organized on the same day by the field-trained healthcare staff to interview and verify disease eligibility criteria, to complete a disease investigation questionnaire and to collect biological specimens. A nasopharyngeal swab, throat swab, and sputum specimen (in cases with productive cough) were collected following a standard protocol.

A follow-up questionnaire was administrated to all ILI cases approximately 60 days following the disease onset in order to identify any sequelae, hospitalizations, and disease outcome.

The patient identification number and the disease investigation number were associated by a relational database and pre-printed labels that were placed on the questionnaire and any specimen collected.

### Laboratory diagnosis of biological specimens

Nasopharyngeal swabs were tested using the technology of FTD Respiratory pathogens 21 Plus for Real-Time PCR (RT-PCR) detection of influenza A, influenza A(H1N1)pdm09, influenza B, rhinovirus, human coronaviruses NL63, 229E, OC43 and HKU1; parainfluenza viruses 1, 2, 3 and 4; HMPV A/B, HboV, respiratory syncytial virus A/B, adenovirus, enterovirus, parechovirus, *Mycoplasma pneumoniae*, *Chlamydia pneumoniae*, *Staphylococcus aureus*, *Streptococcus pneumoniae* and *Hemophilus influenzae* type B. Considering that the FTD panel did not include the influenza A subtype H3N2, samples that tested positive for influenza A were subsequently tested for this subtype using a real-time PCR assay (abTESTM Flu 4 qPCR I kit (50) (v2.0), AITbiotech).

PCR positive cases of *Streptococcus pneumoniae* were serotyped for 40 different serotypes by using a novel multiplex real-time PCR assay [19]. This method identifies the following serotypes: 1, 2, 3, 4, 5, 6A/B, 6C, 7C, 7F, 8, 9N/L, 9V, 10A, 10F, 11A, 12F, 13, 14, 15A, 15B/C, 16F, 17F, 18C, 19A, 19F, 20, 21, 22F, 23A, 23B, 23F, 24, 31, 33F, 34, 35A, 35B, 35F, 38, 39, and an internal control, LytA.

Throat and sputum specimens underwent bacterial culture according to the standard operating procedures of the Mahosot Hospital Microbiology Laboratory, including culture for *Burkholderia pseudomallei*, and potential pathogens were tested for antibiotic susceptibility by disk diffusion using the methods of the Clinical and Laboratory Standards Institute [20]. Throat swabs were cultured on goat blood agar incubated anaerobically for beta-hemolytic s*treptococci* and on Ashdown agar and broth for *Burkholderia pseudomallei*. Sputum was stained by Gram stain and the Kinyoun method for acid-fast bacilli and cultured on goat blood agar with an optochin disc, chocolate agar and Ashdown agar and broth. Beta-hemolytic *streptococci* were identified to Group level using the Oxoid Streptococcal Grouping Kit (Oxoid, Basingstoke UK). *Streptococcus pneumoniae* was identified by optochin susceptibility and bile solubility, *Hemophilus influenzae* was identified by dependence on X and V factors, *Enterobacteriaceae* were identified by in-house biochemical tests and confirmed by API 20E (BioMerieux, Basingstoke UK) when necessary.

### Data management and statistical analysis

Data from the questionnaire surveys and laboratory analyses were double-entered into a FileMaker Pro (v14) relational database.

An asset-based index for socio-economic status (SES) was developed from a principal component analysis of 27 household variables, including durable assets, water sources, and sanitary systems [21]. Weights for each variable were derived from the factor scores in the first principal component, and households were categorised into quintiles based on their SES score.

Bivariate associations between household-level categorical variables and area (urban, peri-urban, or suburban) were tested using Chi-squared and Fisher’s exact tests. Incidence rates with associated 95% confidence intervals were estimated for the population of metropolitan Vientiane using complex survey analysis methodology [8, 22]. This allowed estimates to be adjusted both for the demographic (age and sex) structure of the Laos urban population (based on the 2015 Population and Housing Census), and for clustering in the survey design by household.

To assess potential risk factors for associations with ILI, we used generalized linear mixed models (GLMMs) adjusting for random effects at household level. Three binary outcome variables were used, based on whether the participant experienced at least one episode of ILI, virus-positive ILI, and bacteria-positive ILI during the surveillance period. Multivariate GLMM models included age group, sex, and SES by default, while other variables were considered for inclusion if they showed associations with *P* <0.1 in the bivariate models. The final multivariate models were then derived through backwards, stepwise elimination of variables which did not retain statistical significance of *P*<0.1 based on ANOVA.

All statistical analyses were carried out using R version 3.3.2.

## Results

### Cohort characteristics

A total of 995 households including 4885 study participants were recruited from across 25 villages in metropolitan Vientiane. Males represented 52.4% of the study population. The median age was 29 years (range: 0–99), with 1022 (20.9%) of participants in the age group 0–14 years, 3594 (73.6%) aged 15–64 years, and 269 (5.5%) >65 years. The mean number of participants per household was 4.9 (range: 1–17). Overall, 36.9% of the study population reported having been vaccinated with trivalent influenza vaccine within 12 months prior to enrolment.

Characteristics of the study households by area are reported in Table 1. Almost all participants (99.6%) belonged to Lao Loum ethnicity. There was no significant association between the number of children in the household and study area/zone. SES category and education level of the head of household were significantly associated with the living area. (S1 Table for full results of the PCA analysis used to derive the asset-based SES index).

**Table 1:** Characteristics of study households by area.

	No. (%) of study households					
Variable	Overall	Urban	Peri-urban	Suburban	Chi sq	P-value
***N***	995	247	366	382		
**Ethnicity**						
**Hmong**	2 (0.2)	0 (0.0)	1 (0.3)	1 (0.3)	4.11	0.662
**Khmu**	1 (0.1)	0 (0.0)	1 (0.3)	0 (0.0)		
**Lao Loum**	991 (99.6)	247 (100.0)	363 (99.2)	381 (99.7)		
**Mixed**	1 (0.1)	0 (0.0)	1 (0.3)	0 (0.0)		
**SES category**						
**Lowest**	199 (20.0)	62 (25.1)	45 (12.3)	92 (24.1)	53.07	<0.001***
**Low**	199 (20.0)	51 (20.6)	61 (16.7)	87 (22.8)		
**Medium**	199 (20.0)	42 (17.0)	73 (19.9)	84 (22.0)		
**High**	200 (20.1)	39 (15.8)	85 (23.2)	76 (19.9)		
**Highest**	198 (19.9)	53 (21.5)	102 (27.9)	43 (11.3)		
**Education level of head of household**						
**No school**	54 (5.4)	19 (7.7)	16 (4.4)	19 (5.0)	30.20	<0.001***
**Primary**	236 (23.7)	74 (30.0)	67 (18.3)	95 (24.9)		
**Secondary**	246 (24.7)	59 (23.9)	98 (26.8)	89 (23.3)		
**High school**	196 (19.7)	47 (19.0)	61 (16.7)	88 (23.0)		
**University**	263 (26.4)	48 (19.4)	124 (33.9)	91 (23.8)		
**Number of children (0–14 years) in household**						
**0–1**	718 (72.2)	182 (73.7)	249 (68.0)	287 (75.1)	5,07	0,079
**+2**	277 (27.8)	65 (26.3)	117 (32.0)	95 (24.9)		

### Incidence of influenza like illness in the study area

Throughout the surveillance period, a total of 548 episodes of ILI were reported among 476 (9.7%) individuals from 330 (33.2%) households. The estimated incidence of ILI for the metropolitan Vientiane population, adjusted for demographic structure within the study area, was 10.7 (95%CI: 9.4–11.9) episodes per 100 person-years. On average, each participant reported 0.11 (range: 0–3) episodes of ILI, with a mean of 0.55 (range: 0–8) episodes per household. Fever and cough were the two commonest symptoms, reported by 92.0% and 89.6% of cases respectively. Sore throat, headache and myalgia were reported by over 60% of cases.

The results of the multivariate models for each of ILI outcomes are presented in Table 2. Variables were considered for inclusion if they showed associations with P <0.1 in the bivariate models (S2 Table). Males and young adults (25–44 years) were significantly less likely to report ILI. The number of bedrooms was inversely associated with ILI (adjusted odds ratio, AOR 0.89 per room increase; 95% CI: 0.79–1.00) and bacteria-positive ILI (AOR: 0.82; 95% CI:0.67–0.99). Having a pre-existing chronic condition was significantly associated with ILI (AOR: 1.43; 95% CI: 1.01–2.09) and virus-positive ILI (AOR:1.78; 95%CI: 1.15–2.77) but not with bacteria-positive ILI. Meanwhile, low SES and recent history of influenza vaccination were significant predictors for bacteria-positive ILI, but not ILI or virus-positive ILI.

**Table 2:** Multivariate analyses for associations with reporting of at least one episode of ILI, virus-positive ILI, and bacteria-positive ILI.

	ILI			Virus-positive ILI			Bacteria-positive ILI		
	AOR	(95% CI)	P-value	AOR	(95% CI)	P-value	AOR	(95% CI)	P-value
**Male sex**	0.63	(0.50–0.78)	<0.0001	0.61	(0.46–0.81)	0.001	0.74	(0.51–1.07)	0.11
**Age group (ref: 15 to 24)**									
**0 to 4**	2.64	(1.63–4.27)	<0.0001	3.55	(1.99–6.34)	<0.0001	5.17	(2.48–10.8)	<0.0001
**5 to 14**	2.44	(1.68–3.56)		2.03	(1.24–3.32)		4.47	(2.42–8.24)	
**25 to 34**	0.77	(0.51–1.17)		0.82	(0.48–1.41)		0.85	(0.40–1.83)	
**35 to 44**	1.01	(0.66–1.54)		1.08	(0.62–1.85)		1.17	(0.55–2.52)	
**45 to 64**	2.21	(1.56–3.13)		1.96	(1.24–3.10)		2.09	(1.11–3.95)	
**65 +**	2.15	(1.30–3.56)		1.52	(0.77–3.00)		2.45	(1.00–6.03)	
**SES category (ref: Lowest)**									
**Low**	0.9	(0.59–1.38)	0.13	1.08	(0.64–1.85)	0.59	0.71	(0.38–1.34)	0.002
**Medium**	0.87	(0.57–1.34)		1.01	(0.59–1.73)		0.48	(0.24–0.93)	
**High**	0.60	(0.38–0.94)		0.76	(0.43–1.36)		0.25	(0.11–0.55)	
**Highest**	0.99	(0.62–1.58)		1.18	(0.66–2.12)		0.58	(0.28–1.21)	
**No. of bedrooms**	0.89	(0.79–1.00)	0.04	0.89	(0.77–1.02)	0.10	0.82	(0.67–0.99)	0.04
**Pre-existing chronic condition**	1.45	(1.01–2.09)	0.04	1.78	(1.15–2.77)	0.01	n/a	-	-
**Smoker**	n/a	-	-	n/a	-	-	0.44	(0.18–1.08)	0.07
**Flu Vaccination in past 12 months**	n/a	-	-	n/a	-	-	1.69	(1.10–2.58)	0.02

During the follow-up period, 3 cases (0.5%) were hospitalized for their respiratory illness. Two had tested positive for Coronavirus 63, from the disease investigation (i.e. prior to their hospitalization), while in the third patient no pathogens were detected. All 548 cases were alive at the point of follow-up.

### Etiological profile

Among the 548 nasal swabs, the FTD PCR assay detected at least one potential pathogen in 65.1% of specimens, with 50.7% of nasal swabs testing positive for a least one virus, and 28.8% positive for at least one bacterium. Throat swabs were obtained from 534 (97.4%) cases and underwent microbiological culture. Sputum specimens of sufficient quality for microbiological testing were also obtained from 37 (6.8%) of the cases (a further 20 sputum samples received were rejected as salivary on the basis of microscopy for epithelial and white cells and were not processed).

### Bacterial profile

**Fig 3:**
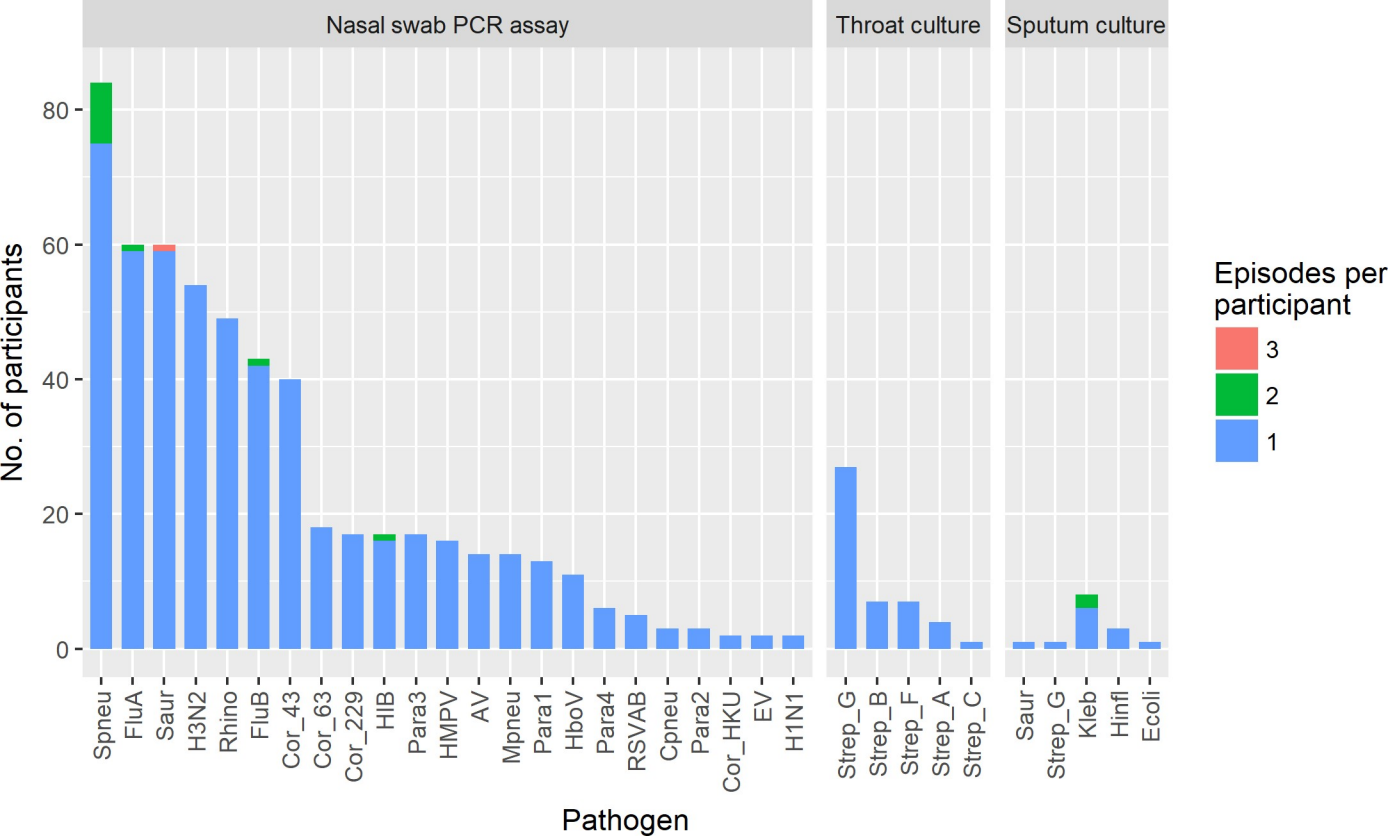
Frequency of detection of respiratory pathogens among study participants.

The most common bacteria detected by real-time PCR in nasal swabs were *Streptococcus pneumoniae* (93 episodes, S3 Table for details on *Streptococcus pneumoniae* serotypes), followed by *Staphylococcus aureus* (62 episodes) (Fig 3).

Culture growth of a potential bacterial pathogen was observed in 46 (8.6%) and 15 (40.5%) of throat and sputum samples, respectively. *streptococcus* were the only potential pathogens isolated from throat swabs (4 Group A, 7 Group B, 1 Group C, 7 Group F and 27 Group G, based on Lancefield classification). *Enterobacteriaceae* (mainly *Klebsiella pneumoniae*) accounted for the majority (11) of isolates reported from sputum samples (together with *Staphylococcus aureus* in one sample), with three growing *Haemophilus influenzae* and one a Group G *streptococcus* (which was also isolated from the same patient’s throat swab). However, the clinical significance of many of isolates is questionable. Interestingly, none of the sputum samples grew *Streptococcus*. *pneumoniae* and *Burkholderia pseudomallei* and acid-fast bacilli were not detected from any sample.

### Viral profile

Influenza A (61 episodes), rhinovirus (49 episodes), influenza B (44 episodes), and coronavirus OC43 (40 episodes) were the main virus isolated from the nasal swabs tested by FTD (Fig 3). Of the 61 nasal swabs positive for influenza A, 54 (88.5%) and 2 (3.3%) tested positive for subtypes A/H3N2 and A/H1N1-pdm09, respectively. A total of 39 parainfluenza virus cases were detected, among which all four types were observed, with type 3 the most common (17 cases, 43%), followed by type 1 (13 cases, 33.3%).

### Co-infections and temporal patterns

More than one potential pathogen was detected in 24.1% (132/548) of ILI episodes. A wide range of combinations of co-infecting agents was observed, although *Streptococcus pneumoniae* was the most frequently detected agent in co-infections. Co-detection of *Streptococcus pneumoniae* with influenza (12 cases with influenza B and 6 cases with influenza A), *Staphylococcus*. *aureus* (11 cases), and *Hemophilus influenzae* type B (11 cases) were among the most common. *Streptococcus pneumoniae* detection was significantly correlated with *Hemophilus influenzae* type B detection (Spearman’s rho = 0.22; *P*<0.001) but not with influenza viruses or *Staphylococcus*. *aureus* (S1 and S2 Figs).

**Fig 4:**
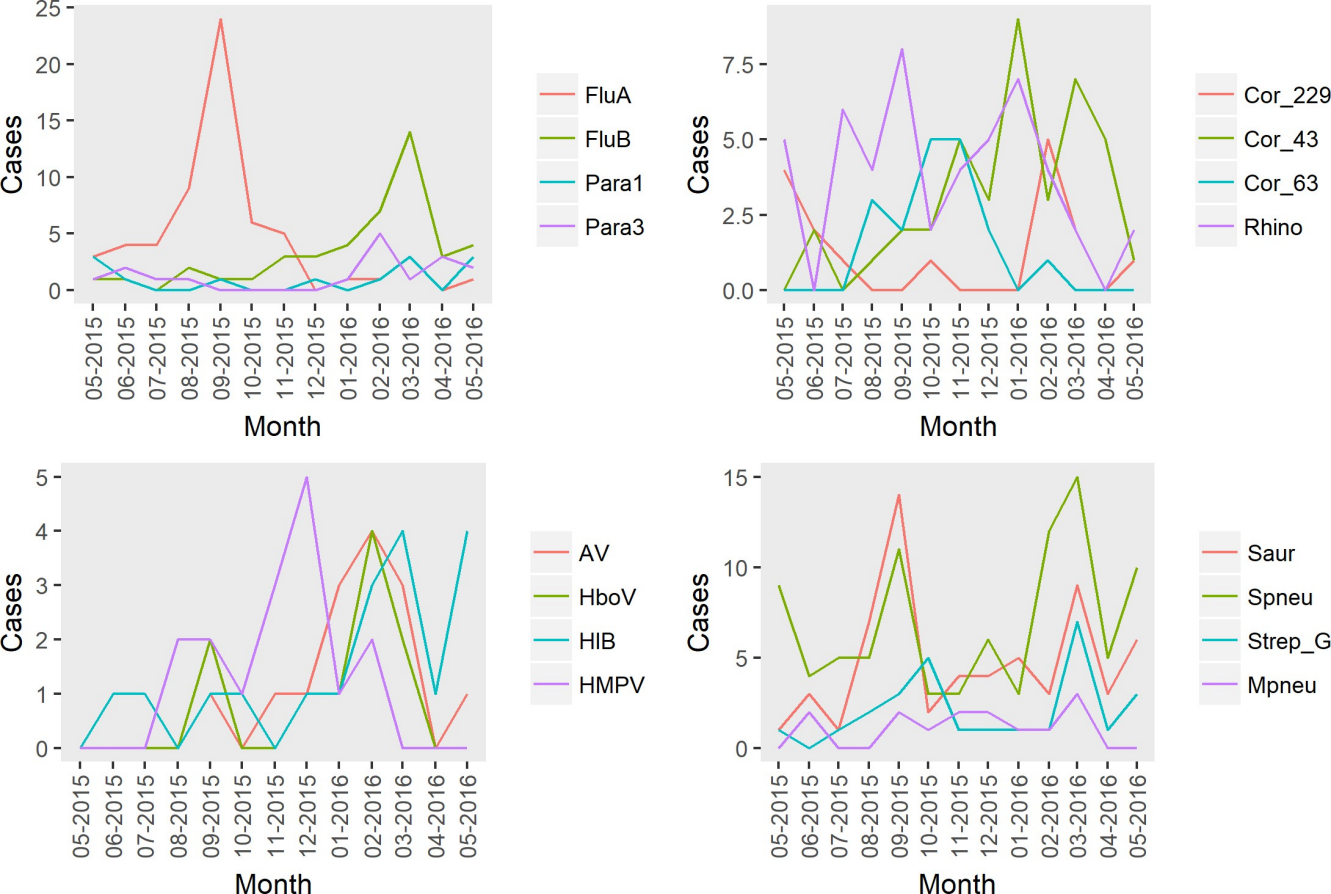
Number of cases by month for the 16 most commonly detected pathogens.

We observed the presence of temporal patterns for the studied respiratory pathogens (Fig 4). Influenza A showed a clear peak between July-October, while Influenza B, coronaviruses, and parainfluenza viruses, tended to be detected more frequently from January through March. Peaks in detection of potentially significant bacteria tended to coincide with these peak periods of viral activity.

### Incidence estimates by pathogen

**Fig 5:**
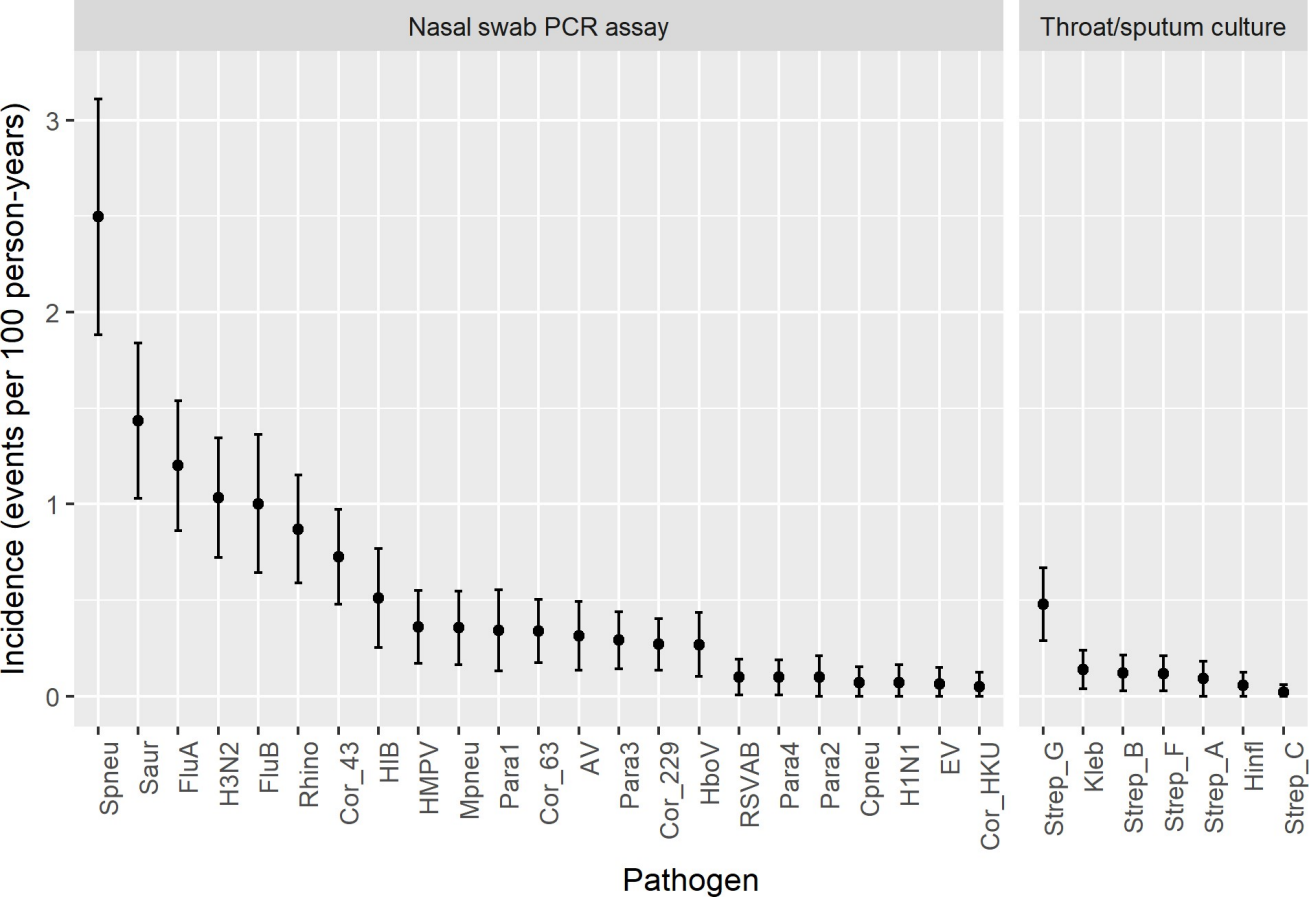
Estimated incidence for respiratory pathogens in metropolitan Vientiane.

Estimated incidence rates (events per 100 person-years) and 95% confidence intervals for each pathogen, adjusted for the demographic structure of the Lao urban household population and clustering of the survey design, are presented in Fig 5 (S4 Table for incidence estimates). The highest incidence rates were found for *Streptococcus pneumoniae* (2.5, 95%CI: 1.88–3.11) followed by *Staphylococcus aureus* (1.44, 95%CI: 1.03–1.84), influenza A (1.20, 95%CI: 0.86–1.54) and B (1.00, 95%CI: 0.64–1.36), and rhinovirus (0.87, 95%CI: 0.59–1.15) (S4 Table for incidence estimates).

**Fig 6:**
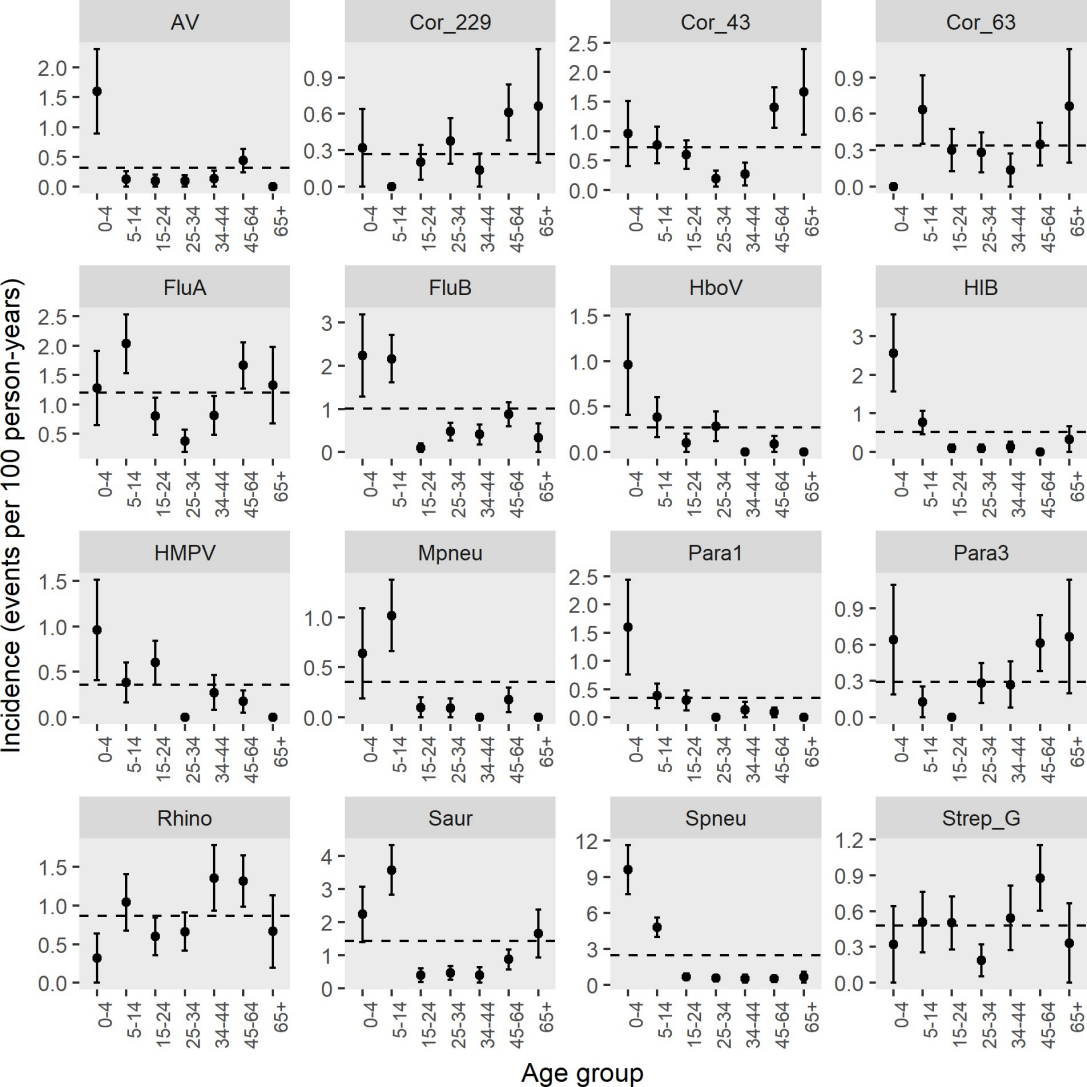
Incidence of the most commonly detected pathogens by age-group. Error bars represent -/+ standard errors. Dashed horizontal lines represent the age and sex adjusted estimate for the overall Vientiane capital population.

Incidence rate by age-groups are shown in Fig 6. *Streptococcus pneumoniae*, influenza B, parainfluenza 1, HMPV, HboV, adenovirus, and *Hemophilus influenzae* type B detection rates were distinctly highest in the youngest (0–4 years) age group while the incidence of influenza A, coronavirus NL63, *Staphylococcus aureus*, and *Mycoplasma pneumoniae* were highest among school-aged (5–14 years) children. The highest incidence rate for coronaviruses was seen in those older than 45 years.

### Symptoms associated with pathogen-specific diagnosis

Analysis of symptoms in relation to specific pathogens detected among cases revealed a number of significant associations, although their diagnostic value was generally limited as indicated by low positive predictive values (S5 Table). Measured fever was of reasonable specificity (>70%) for laboratory confirmed influenza and *Streptococcus pneumoniae*, while myalgia and sore throat were more sensitive (>80%) but less specific (<40%) indicators for influenza. Cyanosis was of high estimated specificity (95%CI: 98–100%) but very low sensitivity (95%CI: 1–10%) for *Streptococcus pneumoniae*. Chills was of high specificity (95% CI: 88–93%) but uncertain sensitivity (95% CI: 9–99%) due to the low number of cases for detection of *H*. *influenzae* through microbial culture, although this symptom was not significantly associated with *Hemophilus influenzae* type B detection by PCR. Other positive symptomatic associations included headache with rhinovirus, and myalgia with coronavirus.

### Temporal clustering of infections by household

To examine potential disease transmission within households, temporal clusters of infections were identified, with a cluster defined as two or more episodes of the same pathogen detected in the same household with onset of symptoms within 21 days of each other. Clusters involving *Streptococcus pneumoniae* were included only if the same serotype was detected among all cases within the clusters. Clusters of *Staphylococcus aureus* infection (for which only one cluster was observed) were excluded as typing of *Staphylococcus aureus* isolates was not conducted.

Overall, 14 clusters were identified (Table 3). The interval period for the onset of secondary cases within each cluster was less than 10 days in all but one cluster (18 days). Five clusters were due to influenza A/H3N2 virus, four were due to influenza B, while Parainfluenza 1, Parainfluenza 4, Rhinovirus and HMPV were each associated with a single cluster. Common *Streptococcus pneumoniae* genotypes were detected in two clusters. Across these 14 household clusters, the “index cases” (i.e. the cases with the earliest date of symptom onset) were children (<18 Y) in four clusters, and adults (≥18 Y) in eight clusters. In two clusters, the index cases involved both a child and an adult with the same date of symptom onset.

**Table 3:** Temporal clustering of infections within households. Table shows cases with the same pathogen detected that occurred within the same household within 21 days of each other.

Household ID	Shared infection(s)	Cases in cluster (position in household^a^, age in years)			Interval between onset of cases (days)
		First	Second	Third	
**10–275**	FluA/H3N2	Daughter, 16 ^b^ Wife, 46 ^b^	-	-	0
**12–900**	FluA/H3N2	Wife, 47	Wife, 48	-	3
**25–226**	FluA/H3N2	Granddaughter, 3 ^b^ Daughter, 13 ^b^	-	-	0
**17–586**	FluA/H3N2	Daughter, 16	Son, 3	-	18
**18–663**	FluA/H3N2	Head (male), 64	Grandson, 6		3
**07-049/1**	FluB	Granddaughter, 18	Daughter, 2	-	1
**07-321/1**	FluB	Head (male), 48	Head (female), 39	-	6
**21–092**	FluB	Daughter, 4	Daughter, 22	-	3
**11–266**	FluB and *S*. *pneumoniae* (16F)	Daughter, 12	Wife, 58	Head (male), 60	1,2
**02–220**	HMPV	Head (female), 43	Son, 5	-	8
**25–114**	Para1	Wife, 45	Granddaughter, 2	-	2
**24–010**	Para4	Son, 25	Wife, 37	-	9
**23–065**	Rhino	Head (male), 44 ^b^ Mother, 99 ^b^	-	-	0
**13–120**	*S*. *pneumoniae* (19F)	Son, 14 ^b^ Son, 22 ^b^		-	0

a Position in household in relation to head of household

b Cases within a cluster which shared the same date of onset

### Antimicrobial susceptibility

Overall, 72.3% (34/47) of *streptococcus* strains were resistant to tetracycline while none showed resistance to ofloxacin. The majority of *Klebsiella* sp. cultures (8/9 cultures; 88.9%) were resistant to ampicillin. Of three *H*. *influenzae* cultures tested, 66.7% were resistant to ampicillin (S6 Table).

## Discussion

Respiratory infections represent one of the main causes of morbidity and mortality in the general population [23]. Community-based studies carried out in high income countries have provided key insights on the epidemiology and etiology of these infections and on their burden among children who are also considered as the main source of transmission within the household [24,25]. However, fewer data are available from low and middle-income countries.

Establishing the etiology of ILI in the general population is important in order to identify the importance of each pathogen, improve management and guide antiviral treatment to limit the emergence and spread of antibiotic resistance [26]. However, little has been published from South-East Asia regarding viral or bacterial pathogens that can contribute to ILI in a community setting. To the best of our knowledge, this is the first community-based surveillance that prospectively monitored the incidence of ILI and its causative pathogens in Vientiane capital in Lao PDR.

A higher incidence of respiratory infections in lower SES households have been reported in several studies and is thought to be related to overcrowding, poor environmental sanitation and poor hygienic practices [27]. Our results are consistent with these previous studies, with a lower rate of ILI observed among Vientiane households in higher SES categories and with a larger number of bedrooms.

Contrary to temperate countries where there is a clear peak of respiratory virus activity during winter, the seasonal activity of these viruses is not so well defined in tropical countries. Two annual peaks, a single annual peak or no distinct seasonality for influenza virus have been reported in the tropics [28–30]. A single peak of influenza activity during the rainy season has been reported in Cambodia [31] and Thailand [32]. In our study, influenza viruses were the most common viral respiratory pathogens followed by rhinovirus. The distribution of influenza virus was marked by two distinct peaks, dominated by influenza A strains (mainly A/H3N2) from July to October and by influenza B strains from February to April. The year-round ILI activity in Vientiane Capital reported by Kieffer et al. [16] was also observed in our study. However, given that our study only covered a single 12-month period, it would not be appropriate to infer whether the observed data are representative of seasonal patterns in Vientiane more generally.

Over the course of the study i.e. 2015–2016, 9.7% of the study population experienced at least one episode of ILI. In a hospital-based study carried out in Lao PDR, ILI cases accounted for 10% of all patients presenting to seven government-run hospital out-patient departments and emergency rooms in Vientiane Capital from 2008 to 2010 [33]. Our estimate of ILI incidence of 0.11 episodes per person-year is very similar to the rate (0.12 per person year) reported in a household surveillance study in Vietnam [34]. Consistent with what has been reported in the literature [35–37], we observed the highest rates of ILI among children (<15years) and older adults (>45 years).

A known virus or bacterium was identified by the PCR-based assay in 65.1% of the 548 nasal swabs samples, and by microbial culture in 8.6% and 45.9% of throat and sputum specimens respectively. However, the clinical significance of the bacteria was frequently difficult to establish. Co-detection was identified in 24.1% of ILI cases, with influenza (A and B) and *Streptococcus pneumoniae* being the most common combination. Recently, new evidence has emerged regarding an interaction between these two pathogens. Some authors believe that influenza induces susceptibility to secondary pneumococcal infections while others reject the unidirectional effect of the virus on *Streptococcus pneumoniae* by arguing that asymptomatic pneumococcal carriage may be an indicator of subsequent influenza virus infection ([38] and references therein). It is also important to note that we did not detect a statistically significant association between *Streptococcus pneumoniae* and influenza; thus, the relatively high frequency of these co-infections does not necessarily support hypotheses for influenza-induced susceptibility to pneumococcal infection (or vice versa). We did, however, observe a significant association between *Streptococcus pneumoniae* and *H*. *influenzae* infection. A synergistic association between these agents has previously been observed in children [39], and also in an experimental study on Chinchillas [40], with the latter suggesting that *H*. *influenzae* co-infection promotes pneumococcal biofilm formation and persistence.

Interestingly, we found that recent history of influenza vaccination was associated with a relatively small but significant increase in risk of a bacteria-positive ILI episode, even after adjusting for other variables (AOR 1.69; 95% CI: 1.10–2.58). A previous *in vivo* study found that live attenuated influenza vaccine (LAIV) promotes colonisation by *Streptococcus pneumoniae* and *Staphylococcus aureus* of the upper respiratory tracts of mice [41], although the clinical relevance of this finding has been disputed by others, given that previous studies reported no such association between LAIV and nasal carriage of bacteria in humans ([42] and references therein). One other observation of note was the complete absence of *B*. *pseudomallei*, which has previously been reported to cause a mild, self-limiting ‘flu-like illness in northern Australia [43], in this population, despite the fact that melioidosis is common in the Vientiane area [44].

Viral etiologies in the present study were similar to those found in other studies of ILI or acute respiratory illness in Asia [34], Australia [45] and the US [46]. Influenza virus was the most common virus present in 11% of ILI swabs, followed by rhinovirus and coronavirus.

Incidence profiles by age-group varied between pathogens. Influenza was the most commonly isolated virus in children 0–14 years old. This is in contrast with hospital-based studies in Asia that reported RSV as the commonest viral pathogen detected among young children with acute respiratory infection [12, 47–49]. This finding supports previous observations that etiological studies carried out in hospital settings may not accurately reflect the situation in the community, as the former are skewed toward more severe cases. We found the incidence of influenza B to be particularly high in children compared to other age groups. No obvious pattern regarding the frequency of influenza B and age was observed in a systematic literature review that assessed the global burden of influenza B [50]. As reported by others [34, 46], coronaviruses were common amongst older individuals.

*Streptococcus pneumoniae* and *Staphylococcus aureus* were the main bacteria found in children 0–4 and 5–14 years old respectively. These potential pathogens are also carried by healthy individuals as commensals with varied rates according to the age. The highest rates of *Streptococcus pneumoniae* carriage are in general seen at 6 months to 3 years of age [51,52] and an inverse relationship has previously been observed between the carriage of *Streptococcus pneumoniae* and *Staphylococcus aureus* [53–55]. This finding together with the fact that nasal carriage of these pathogens is known to be the first step of infection [56, 57] could explain the observed differences in the rates of *Streptococcus pneumoniae* and *Staphylococcus aureus* detection in these age groups. *Streptococcus pneumoniae* was the principal bacterial pathogen in recent studies of pneumonia in children in Nepal [58], in India [59] and in the WHO Western Pacific region [60].

Management of respiratory diseases caused by bacteria can be complicated by the presence of resistant strains. We found that except streptococcus groups C and F, the majority of isolated streptococcus strains were resistant to tetracycline. High rates of resistance to tetracycline were also found in a study of *Streptococcus pneumoniae* isolated in China [61], Korea [62] and Vietnam [63]. On the contrary, none of the cultured streptococcus isolates in this work were resistant to ofloxacin.

Although the incidence of many respiratory pathogens was highest in children, our study did not provide any evidence that children contribute significantly more to household transmission compared with other age groups. None of the three main ILI outcome variables examined (ILI, virus-positive ILI or bacteria-positive ILI) were significantly associated with the number of children living in the household, in bivariate or multivariate analyses. Furthermore, in the 14 potential household transmission events identified, the “index” cases (based on date of symptom onset) were children in less than half of the clusters. These findings are consistent with those of the aforementioned household cohort study in Vietnam, which similarly found little evidence to suggest that children are the main contributors to household transmission [34]. In slight contrast to the Vietnam study, however, we observed household clusters to be most frequently associated with influenza rather than rhinovirus.

Nonetheless, the limitations of community-based ILI surveillance for detecting household transmission events should be acknowledged. For example, this approach relies on households to report ILI symptoms when contacted by the surveillance team, and reporting rates may vary by age and severity of symptoms. Some asymptomatic or mild cases are likely to be missed, and specimen sampling will not always coincide with the optimum time window for pathogen detection. In addition, self-reported date of symptom onset may not be an accurate proxy for identifying primary and secondary cases within household clusters. Longitudinal serological surveys are needed to more accurately infer household transmission risk, although this requires considerable additional resources and to date such serological surveys have tended to focus on a single pathogen such as influenza [64]. *Hemophilus influenzae* is among the leading bacterial causes of respiratory infection. Except *Hemophilus influenza* B, our study did not include other serotypable *Hemophilus influenza* strains due to limited available funds. Nasal samples are however stored in a biobank and will be the subject of further analyses.

Another limitation of our study is that *Staphylococcus aureus* and *Streptococcus pneumoniae* are commensal bacteria. As a consequence, the high rate detection of these bacteria in our study could reflect the carriage in the study population, independently of ILI. Also, while the community-based design is appropriate for identifying and estimating the incidence of circulating respiratory pathogens, hospital-based surveillance is better powered for identifying the causative agents in more severe clinical cases.

Despite these limitations, our study has provided comprehensive insights on the etiology and incidence of ILI in Laos and contributes to the limited but growing evidence on the epidemiology and burden of respiratory pathogens in low-income, tropical countries. The major strength of this study is that it considered a much more comprehensive range of pathogens, including both bacterial and viral agents, than is typically covered in ILI surveillance studies, particularly in developing country settings. The study could also have some direct benefits for the study population. For example, it may lead some participants to seek medical attention more often than they normally would, thus benefiting participants by increased medical attention. Second, public health interventions and measures may be influenced by the results of this study through a better understanding of the etiology of respiratory disease in this setting.

In summary, LaCoRIS has generated valuable data on respiratory disease burden, patterns of etiologies associated with community-acquired acute respiratory illness, and data on *Streptococcus pneumoniae* serotypes in Laos. The accumulating data on household-level transmission, and the biobank repository of specimens constitutes an important resource to facilitate future investigations on influenza epidemiology. Establishment of a surveillance strategy in Laos to monitor trends in the epidemiology and burden of acute respiratory infections, and for early detection of emerging pathogens, is required to minimize their impact on human health.

## Supporting information

S1 Table Socio Economic Status. (DOCX)

S2 Table Bivariate analyses for risk factors associated with experiencing at least one episode of ILI, virus-positive ILI, and bacteria-positive ILI. Results are derived from generalised linear mixed models adjusting for random effects at household level.
(DOCX)

S3 Table Streptococcus pneumoniae Typing. (DOCX)

S4 Table Number of episodes and estimated incidence for respiratory pathogens in metropolitan Vientiane. Incidence estimates are adjusted for the demographic structure of the Laos urban household population in 2015.
(DOCX)

S5 Table Analysis of association between symptoms and pathogens. (DOCX)

S6 Table Frequency of antimicrobial resistance among bacterial cultures from throat and sputum specimens. (DOCX)

S1 Fig Frequency of co-infections among 548 ILI case investigations. The number and color within each square represent the number of co-infections involving each pairwise combination of pathogens.
(TIF)

S2 Fig Correlation matrix of co-infections among 548 ILI case investigations. The color within each square represents the Spearman correlation coefficient (red is negative, blue is positive). Symbols *, **, and *** indicated statistically significant correlation at *P*<0.05, <0.01, and <0.001, respectively. Correlation coefficients could not be calculated between enterovirus (EV) and cultured pathogens (*Klebsiella pneumonia*, *Haemophilus influenzae* and *Streptococcus*), as microbial culture tests were not conducted for the single EV positive case.
(TIF)
