# Correction: "Epidemiology and aetiology of influenza-like illness among households in metropolitan Vientiane, Lao PDR": A prospective, community-based cohort study

**DOI:** 10.1371/journal.pone.0216491

**Published:** 2019-04-30

**Authors:** James W. Rudge, Nui Inthalaphone, Rebecca Pavlicek, Phimpha Paboriboune, Bruno Flaissier, Chou Monidarin, Nicolas Steenkeste, Viengmon Davong, Manivanh Vongsouvath, K. A. Bonath, Melinda Messaoudi, Mitra Saadatian-Elahi, Paul Newton, Hubert Endtz, David Dance, Glaucia Paranhos Baccala, Valentina Sanchez Picot

There is an error in affiliation 7 for authors Viengmon Davong, Manivanh Vongsouvath, Paul Newton, and David Dance. The correct affiliation 7 is: Lao-Oxford-Mahosot Hospital-Wellcome Trust Research Unit, Vientiane, Laos.

## References

[1] Rudge JW, Inthalaphone N, Pavlicek R, Paboriboune P, Flaissier B, Monidarin C, et al (2019) “Epidemiology and aetiology of influenza-like illness among households in metropolitan Vientiane, Lao PDR”: A prospective, community-based cohort study. PLoS ONE 14(4): e0214207 10.1371/journal.pone.0214207 30951544 PMC6450629

