# Supplementary material for: “Epidemiology and aetiology of influenza-like illness among households in metropolitan Vientiane, Lao PDR”: A prospective, community-based cohort study
Source: PLoS One. 2019 Apr 5;14(4):e0214207. doi: 10.1371/journal.pone.0214207 (PMC6450629; doi:10.1371/journal.pone.0214207)
Supplement: S1 Table — (DOCX) [file pone.0214207.s001.docx]

**S1 Table:** Socio Economic Status

SES category was based on a principal component analysis (PCA) of 27 household variables, including durable assets, water sources, and sanitary systems. The first principal component used for the SES index accounted 7.6% of variation in asset ownership across households. Assets with the highest factor scores (>0.3) in the first principal component (and therefore most strongly weighted in the SES index), were car, gas stove, air conditioning, water cooler, and modern flush toilet.

| Asset | Mean | St dev | Factor score^a^ |
| --- | --- | --- | --- |
| Electricity | 0.997 | 0.055 | 0.035 |
| Radio | 0.156 | 0.363 | 0.150 |
| Telephone | 0.978 | 0.147 | 0.009 |
| Television | 0.982 | 0.133 | 0.036 |
| Tape recorder | 0.090 | 0.287 | 0.196 |
| Bicycle | 0.382 | 0.486 | 0.110 |
| Motorcycle | 0.963 | 0.189 | 0.051 |
| Car | 0.445 | 0.497 | 0.327 |
| Tuk tuk | 0.024 | 0.154 | 0.045 |
| Tec Tec (iron buffalo) | 0.006 | 0.077 | 0.037 |
| Refrigerator | 0.967 | 0.179 | 0.052 |
| Charcoal stove | 0.897 | 0.303 | -0.135 |
| Gas stove | 0.361 | 0.480 | 0.323 |
| Electric stove | 0.411 | 0.492 | 0.241 |
| Electric rice mill | 0.968 | 0.177 | 0.073 |
| Watercooler | 0.635 | 0.482 | 0.304 |
| Fan | 0.991 | 0.095 | 0.009 |
| Air conditioner | 0.441 | 0.497 | 0.376 |
| Washing machine | 0.745 | 0.436 | 0.290 |
| Animals | 0.483 | 0.500 | 0.048 |
| Water sources: |  |  |  |
| Bottled water | 0.978 | 0.147 | -0.014 |
| Tap | 0.463 | 0.499 | -0.021 |
| Well | 0.018 | 0.133 | -0.050 |
| Sanitary system |  |  |  |
| Modern flush toilet | 0.518 | 0.500 | 0.391 |
| Toilet without septic tank | 0.486 | 0.500 | -0.381 |
| Toilet with septic tank | 0.010 | 0.100 | 0.038 |
| Pit | 0.003 | 0.055 | -0.016 |

^a^ Factor scores derived from the first principal component, used as weights for each asset when calculating the socioeconomic score for each household.
