# Supplementary material for: “Epidemiology and aetiology of influenza-like illness among households in metropolitan Vientiane, Lao PDR”: A prospective, community-based cohort study
Source: PLoS One. 2019 Apr 5;14(4):e0214207. doi: 10.1371/journal.pone.0214207 (PMC6450629; doi:10.1371/journal.pone.0214207)
Supplement: S2 Table — Results are derived from generalised linear mixed models adjusting for random effects at household level. (DOCX) [file pone.0214207.s002.docx]

**S2 Table:** Bivariate analyses for risk factors associated with experiencing at least one episode of ILI, virus-positive ILI, and bacteria-positive ILI. Results are derived from generalised linear mixed models adjusting for random effects at household level

|  | ILI |  |  | | Virus-positive ILI | |  |  | Bacteria-positive ILI |  |
| --- | --- | --- | --- | --- | --- | --- | --- | --- | --- | --- |
|  | **OR (95% CI)** | ***P*** |  | | **OR (95% CI)** | | ***P*** |  | **OR (95% CI)** | *P* |
| Individual level variables: |  |  |  | |  | |  |  |  |  |
| Male sex | 0.65 (0.53,0.80) | <0.0001 |  | | 0.64 (0.48,0.84) | | 0.001 |  | 0.69 (0.49,0.97) | 0.03 |
| Age group (ref: 15 to 24) |  |  |  | |  | |  |  |  |  |
| 0 to 4 | 2.57 (1.59,4.14) | <0.0001 |  | | 3.52 (1.97,6.28) | | <0.0001 |  | 6.36 ( 3.01,13.46) | <0.0001 |
| 5 to 14 | 2.45 (1.69,3.56) |  |  | | 2.11 (1.29,3.44) | |  |  | 5.24 (2.80,9.81) |  |
| 25 to 34 | 0.78 (0.52,1.18) |  |  | | 0.85 (0.50,1.46) | |  |  | 0.81 (0.37,1.74) |  |
| 35 to 44 | 1.10 (0.72,1.68) |  |  | | 1.23 (0.71,2.12) | |  |  | 1.11 (0.51,2.41) |  |
| 45 to 64 | 2.38 (1.69,3.34) |  |  | | 2.30 (1.47,3.59) | |  |  | 1.93 (1.02,3.66) |  |
| 65 + | 2.35 (1.43,3.84) |  |  | | 1.82 (0.94,3.54) | |  |  | 2.13 (0.86,5.24) |  |
| Smoker | 0.65 (0.45,0.94) | 0.02 |  | | 0.60 (0.36,0.99) | | 0.04 |  | 0.27 (0.11,0.62) | 0.002 |
| Flu Vaccination in past 12 months | 1.30 (1.02,1.67) | 0.03 |  | | 1.19 (0.87,1.63) | | 0.27 |  | 2.12 (1.42,3.16) | <0.001 |
| Pre-existing chronic condition | 1.74 (1.24,2.43) | 0.001 |  | | 1.97 (1.31,2.95) | | 0.001 |  | 1.05 (0.58,1.90) | 0.86 |
| Household level variables: |  |  |  | |  | |  |  |  |  |
| Area (ref: urban) |  |  |  | |  | |  |  |  |  |
| Periurban | 0.87 (0.62,1.23) | 0.64 |  | | 1.12 (0.72,1.73) | | 0.63 |  | 0.97 (0.54,1.73) | 0.47 |
| Suburban | 0.99 (0.71,1.39) |  |  | | 1.23 (0.80,1.91) | |  |  | 1.29 (0.73,2.28) |  |
| SES category (ref: Lowest) |  |  |  | |  | |  |  |  |  |
| Low | 0.84 (0.56,1.25) | 0.06 |  | | 1.07 (0.64,1.79) | | 0.61 |  | 0.69 (0.38,1.27) | 0.001 |
| Medium | 0.80 (0.53,1.19) |  |  | | 1.00 (0.60,1.69) | |  |  | 0.45 (0.24,0.86) |  |
| High | 0.53 (0.34,0.81) |  |  | | 0.71 (0.41,1.23) | |  |  | 0.23 (0.11,0.49) |  |
| Highest | 0.79 (0.53,1.19) |  |  | | 0.98 (0.58,1.65) | |  |  | 0.49 (0.26,0.92) |  |
| Education level of head of household  (ref: no school) | | | |  | |  |  |  |  |  |
| Primary | 1.34 (0.68,2.62) | 0.23 |  | | 2.58 (0.95,7.00) | | 0.11 |  | 1.50 (0.47,4.75) | 0.64 |
| Secondary | 1.51 (0.77,2.94) |  |  | | 2.69 (0.99,7.30) | |  |  | 1.56 (0.49,4.95) |  |
| High school | 1.95 (0.99,3.84) |  |  | | 3.49 (1.28,9.52) | |  |  | 2.19 (0.69,6.99) |  |
| University | 1.52 (0.78,2.95) |  |  | | 2.48 (0.92,6.71) | |  |  | 1.64 (0.52,5.15) |  |
| No. of household members | 0.94 (0.89,0.99) | 0.02 |  | | 0.93 (0.87,1.00) | | 0.06 |  | 0.92 (0.84,1.02) | 0.10 |
| No. of sleeping rooms | 0.87 (0.79,0.97) | 0.01 |  | | 0.88 (0.78,1.00) | | 0.05 |  | 0.76 (0.64,0.90) | 0.00 |
| Residents per sleeping room | 1.03 (0.91,1.17) | 0.65 |  | | 1.01 (0.87,1.19) | | 0.87 |  | 1.13 (0.92,1.39) | 0.23 |
| No. of children <15yrs | 0.98 (0.87,1.11) | 0.77 |  | | 0.99 (0.85,1.15) | | 0.88 |  | 1.20 (0.99,1.46) | 0.06 |
| No. of children <6yrs | 0.91 (0.74,1.11) | 0.34 |  | | 1.00 (0.78,1.28) | | 0.98 |  | 1.08 (0.78,1.50) | 0.66 |
| Charcoal stove | 0.81 (0.53,1.25) | 0.35 |  | | 0.76 (0.45,1.29) | | 0.31 |  | 0.92 (0.44,1.91) | 0.83 |
| Charcoal stove inside | 1.17 (0.88,1.56) | 0.29 |  | | 1.16 (0.80,1.66) | | 0.43 |  | 1.44 (0.90,2.30) | 0.13 |
| Keep domestic animals | 1.17 (0.90,1.52) | 0.24 |  | | 1.18 (0.84,1.64) | | 0.34 |  | 1.02 (0.66,1.58) | 0.94 |
| Keep cats | 1.12 (0.64,1.96) | 0.68 |  | | 1.21 (0.61,2.40) | | 0.58 |  | 0.78 (0.29,2.12) | 0.63 |
| Keep dogs | 0.98 (0.75,1.29) | 0.88 |  | | 0.88 (0.62,1.24) | | 0.45 |  | 0.93 (0.59,1.47) | 0.76 |
| Keep goats or sheep | 1.09 (0.07,17.4) | 0.95 |  | | 2.34 (0.12,45.7) | | 0.57 |  | 0.00 (0.00, Inf) | 1.00 |
| Keep chickens | 1.14 (0.82,1.57) | 0.43 |  | | 1.11 (0.74,1.66) | | 0.62 |  | 1.13 (0.66,1.93) | 0.66 |
| Keep ducks | 1.38 (0.86,2.22) | 0.19 |  | | 1.57 (0.88,2.81) | | 0.13 |  | 1.45 (0.66,3.16) | 0.35 |

****P*<0.05; ***P*<0.01; ****P*<0.001**
