# Supplementary material for: “Epidemiology and aetiology of influenza-like illness among households in metropolitan Vientiane, Lao PDR”: A prospective, community-based cohort study
Source: PLoS One. 2019 Apr 5;14(4):e0214207. doi: 10.1371/journal.pone.0214207 (PMC6450629; doi:10.1371/journal.pone.0214207)
Supplement: S3 Table — (DOCX) [file pone.0214207.s003.docx]

**S3 Table:** *Streptococcus pneumoniae Typing*

The 93 *Streptococcus pneumoniae*-positive nasal swabs were subjected to a PCR typing assay which tests for 40 different genotypes. Of 93 *Streptococcus pneumoniae*-positive swabs, 69 (74.2%) tested positive for at least one of these genotypes, with 10 specimens (10.8%) positive for two or more different genotypes. The most commonly detected genotypes were 19F, 6AB, and 14, which were detected in 12 (12.9%), 10 (10.8%) and 7 (7.5%) of *Streptococcus pneumoniae*-positive swabs, respectively.

| *S. pnuemoniae* type | No. positive | (%) |
| --- | --- | --- |
| Total no. of nasal specimens, *N* | 93 |  |
| Unidentified type | 24 | (25.8) |
| 19F | 12 | (12.9) |
| 6AB | 10 | (10.8) |
| 14 | 7 | (7.5) |
| 20 | 5 | (5.4) |
| 3 | 5 | (5.4) |
| 35B | 5 | (5.4) |
| 15BC | 4 | (4.3) |
| 23F | 4 | (4.3) |
| 13 | 3 | (3.2) |
| 15A | 3 | (3.2) |
| 16F | 3 | (3.2) |
| Sg18 | 3 | (3.2) |
| 1 | 2 | (2.2) |
| 10A | 2 | (2.2) |
| 11A | 2 | (2.2) |
| 19A | 2 | (2.2) |
| 34 | 2 | (2.2) |
| 35F | 2 | (2.2) |
| 38 | 1 | (1.1) |
| 12F | 1 | (1.1) |
| 17F | 1 | (1.1) |
| 23A | 1 | (1.1) |
| 4 | 1 | (1.1) |
| 6C | 1 | (1.1) |
| 7C | 1 | (1.1) |
| 9V | 1 | (1.1) |
