# Supplementary material for: “Epidemiology and aetiology of influenza-like illness among households in metropolitan Vientiane, Lao PDR”: A prospective, community-based cohort study
Source: PLoS One. 2019 Apr 5;14(4):e0214207. doi: 10.1371/journal.pone.0214207 (PMC6450629; doi:10.1371/journal.pone.0214207)
Supplement: S4 Table — Incidence estimates are adjusted for the demographic structure of the Laos urban household population in 2015. (DOCX) [file pone.0214207.s004.docx]

**S4 Table:** Number of episodes and estimated incidence for respiratory pathogens in metropolitan Vientiane. Incidence estimates are adjusted for the demographic structure of the Laos urban household population in 2015

|  | **Overall** | **Age group** | | | | | | |
| --- | --- | --- | --- | --- | --- | --- | --- | --- |
|  |  | **0 to 4** | **5 to 14** | **15 to 24** | **25 to 34** | **35 to 44** | **45 to 64** | **65 +** |
|  |  |  |  |  |  |  |  |  |
| No. of individuals | 4885 | 286 | 720 | 914 | 970 | 675 | 1045 | 275 |
| No. of ILI episodes | 548 | 49 | 114 | 68 | 56 | 60 | 158 | 43 |
|  |  |  |  |  |  |  |  |  |
|  | **No. (%) of ILI specimens positive** | | |  |  |  |  |  |
| Nasal swabs |  |  |  |  |  |  |  |  |
| Influenza A | 61 (11.1) | 4 (8.2) | 16 (14) | 8 (11.8) | 4 (7.1) | 6 (10) | 19 (12) | 4 (9.3) |
| Influenza A/H3N2 | 54 (9.9) | 2 (4.1) | 14 (12.3) | 8 (11.8) | 4 (7.1) | 5 (8.3) | 18 (11.4) | 3 (7) |
| Influenza A/H1N1 | 2 (0.4) | 2 (4.1) | 0 (0) | 0 (0) | 0 (0) | 0 (0) | 0 (0) | 0 (0) |
| Influenza B | 44 (8) | 7 (14.3) | 17 (14.9) | 1 (1.5) | 5 (8.9) | 3 (5) | 10 (6.3) | 1 (2.3) |
| Rhinovirus | 49 (8.9) | 1 (2) | 8 (7) | 6 (8.8) | 7 (12.5) | 10 (16.7) | 15 (9.5) | 2 (4.7) |
| Coronavirus 229 | 17 (3.1) | 1 (2) | 0 (0) | 2 (2.9) | 4 (7.1) | 1 (1.7) | 7 (4.4) | 2 (4.7) |
| Coronovirus 63 | 18 (3.3) | 0 (0) | 5 (4.4) | 3 (4.4) | 3 (5.4) | 1 (1.7) | 4 (2.5) | 2 (4.7) |
| Coronovirus HKU | 2 (0.4) | 1 (2) | 0 (0) | 0 (0) | 1 (1.8) | 0 (0) | 0 (0) | 0 (0) |
| Coronovirus 43 | 40 (7.3) | 3 (6.1) | 6 (5.3) | 6 (8.8) | 2 (3.6) | 2 (3.3) | 16 (10.1) | 5 (11.6) |
| Parainfluenza 1 | 13 (2.4) | 5 (10.2) | 3 (2.6) | 3 (4.4) | 0 (0) | 1 (1.7) | 1 (0.6) | 0 (0) |
| Parainfluenza 2 | 3 (0.5) | 2 (4.1) | 1 (0.9) | 0 (0) | 0 (0) | 0 (0) | 0 (0) | 0 (0) |
| Parainfluenza 3 | 17 (3.1) | 2 (4.1) | 1 (0.9) | 0 (0) | 3 (5.4) | 2 (3.3) | 7 (4.4) | 2 (4.7) |
| Parainfluenza 4 | 6 (1.1) | 0 (0) | 0 (0) | 2 (2.9) | 0 (0) | 2 (3.3) | 2 (1.3) | 0 (0) |
| Human Metapneumonia Virus A/B | 16 (2.9) | 3 (6.1) | 3 (2.6) | 6 (8.8) | 0 (0) | 2 (3.3) | 2 (1.3) | 0 (0) |
| Bocavirus | 11 (2) | 3 (6.1) | 3 (2.6) | 1 (1.5) | 3 (5.4) | 0 (0) | 1 (0.6) | 0 (0) |
| Respiratory Syncytial Virus A/B | 5 (0.9) | 1 (2) | 0 (0) | 2 (2.9) | 0 (0) | 0 (0) | 2 (1.3) | 0 (0) |
| Enterovirus | 2 (0.4) | 1 (2) | 1 (0.9) | 0 (0) | 0 (0) | 0 (0) | 0 (0) | 0 (0) |
| Adenovirus | 14 (2.6) | 5 (10.2) | 1 (0.9) | 1 (1.5) | 1 (1.8) | 1 (1.7) | 5 (3.2) | 0 (0) |
| *Staphylococcus aureus* | 62 (11.3) | 7 (14.3) | 28 (24.6) | 4 (5.9) | 5 (8.9) | 3 (5) | 10 (6.3) | 5 (11.6) |
| *Chlamydia pneumoniae* | 3 (0.5) | 0 (0) | 2 (1.8) | 0 (0) | 0 (0) | 0 (0) | 1 (0.6) | 0 (0) |
| *Haemophilus influenzae* type B | 18 (3.3) | 8 (16.3) | 6 (5.3) | 1 (1.5) | 1 (1.8) | 1 (1.7) | 0 (0) | 1 (2.3) |
| *Streptococcus pneumoniae* | 93 (17) | 30 (61.2) | 38 (33.3) | 7 (10.3) | 6 (10.7) | 4 (6.7) | 6 (3.8) | 2 (4.7) |
| *Mycoplasma pneumoniae* | 14 (2.6) | 2 (4.1) | 8 (7) | 1 (1.5) | 1 (1.8) | 0 (0) | 2 (1.3) | 0 (0) |
| Throat/sputum samples |  |  |  |  |  |  |  |  |
| *Klebsiella* | 10 (1.9) | 0 (0) | 0 (0) | 0 (0) | 0 (0) | 2 (3.3) | 5 (3.2) | 3 (7) |
| *Haemophilus influenzae* | 3 (0.6) | 0 (0) | 1 (0.9) | 0 (0) | 0 (0) | 0 (0) | 1 (0.6) | 1 (2.3) |
| *Streptococcus* (any) | 46 (8.7) | 1 (2.7) | 8 (7.3) | 9 (13.2) | 5 (8.9) | 7 (11.7) | 15 (9.5) | 1 (2.3) |
| *Streptococcus* Group A | 4 (0.8) | 0 (0) | 2 (1.8) | 1 (1.5) | 0 (0) | 1 (1.7) | 0 (0) | 0 (0) |
| *Streptococcus* Group B | 7 (1.3) | 0 (0) | 1 (0.9) | 1 (1.5) | 2 (3.6) | 1 (1.7) | 2 (1.3) | 0 (0) |
| *Streptococcus* Group C | 1 (0.2) | 0 (0) | 0 (0) | 1 (1.5) | 0 (0) | 0 (0) | 0 (0) | 0 (0) |
| *Streptococcus* Group F | 7 (1.3) | 0 (0) | 1 (0.9) | 1 (1.5) | 1 (1.8) | 1 (1.7) | 3 (1.9) | 0 (0) |
| *Streptococcus* Group G | 27 (5.1) | 1 (2.7) | 4 (3.7) | 5 (7.4) | 2 (3.6) | 4 (6.7) | 10 (6.3) | 1 (2.3) |
|  |  |  |  |  |  |  |  |  |
|  | **Estimated incidence per 100 person years** | | | | | | | |
| Nasal swabs |  |  |  |  |  |  |  |  |
| Influenza A | 1.20 | 1.28 | 2.03 | 0.80 | 0.38 | 0.81 | 1.66 | 1.33 |
| Influenza A/H3N2 | 1.03 | 0.64 | 1.78 | 0.80 | 0.38 | 0.68 | 1.58 | 1.00 |
| Influenza A/H1N1 | 0.07 | 0.64 | 0.00 | 0.00 | 0.00 | 0.00 | 0.00 | 0.00 |
| Influenza B | 1.00 | 2.24 | 2.16 | 0.10 | 0.47 | 0.41 | 0.88 | 0.33 |
| Rhinovirus | 0.87 | 0.32 | 1.04 | 0.60 | 0.66 | 1.36 | 1.31 | 0.67 |
| Coronavirus 229 | 0.27 | 0.32 | 0.00 | 0.20 | 0.38 | 0.14 | 0.61 | 0.67 |
| Coronovirus 63 | 0.34 | 0.00 | 0.64 | 0.30 | 0.28 | 0.14 | 0.35 | 0.67 |
| Coronovirus HKU | 0.05 | 0.32 | 0.00 | 0.00 | 0.09 | 0.00 | 0.00 | 0.00 |
| Coronovirus 43 | 0.73 | 0.96 | 0.76 | 0.60 | 0.19 | 0.27 | 1.40 | 1.66 |
| Parainfluenza 1 | 0.34 | 1.60 | 0.38 | 0.30 | 0.00 | 0.14 | 0.09 | 0.00 |
| Parainfluenza 2 | 0.10 | 0.64 | 0.13 | 0.00 | 0.00 | 0.00 | 0.00 | 0.00 |
| Parainfluenza 3 | 0.29 | 0.64 | 0.13 | 0.00 | 0.28 | 0.27 | 0.61 | 0.67 |
| Parainfluenza 4 | 0.10 | 0.00 | 0.00 | 0.20 | 0.00 | 0.27 | 0.18 | 0.00 |
| Human Metapneumonia Virus A/B | 0.36 | 0.96 | 0.38 | 0.60 | 0.00 | 0.27 | 0.18 | 0.00 |
| Bocavirus | 0.27 | 0.96 | 0.38 | 0.10 | 0.28 | 0.00 | 0.09 | 0.00 |
| Respiratory Syncytial Virus A/B | 0.10 | 0.32 | 0.00 | 0.20 | 0.00 | 0.00 | 0.18 | 0.00 |
| Enterovirus | 0.06 | 0.32 | 0.13 | 0.00 | 0.00 | 0.00 | 0.00 | 0.00 |
| Adenovirus | 0.31 | 1.60 | 0.13 | 0.10 | 0.09 | 0.14 | 0.44 | 0.00 |
| *Staphylococcus aureus* | 1.44 | 2.24 | 3.58 | 0.40 | 0.47 | 0.41 | 0.88 | 1.66 |
| *Chlamydia pneumoniae* | 0.07 | 0.00 | 0.25 | 0.00 | 0.00 | 0.00 | 0.09 | 0.00 |
| *Haemophilus influenzae* type B | 0.51 | 2.56 | 0.76 | 0.10 | 0.09 | 0.14 | 0.00 | 0.33 |
| *Streptococcus pneumoniae* | 2.50 | 9.60 | 4.83 | 0.70 | 0.57 | 0.54 | 0.53 | 0.67 |
| *Mycoplasma pneumoniae* | 0.35 | 0.64 | 1.02 | 0.10 | 0.09 | 0.00 | 0.18 | 0.00 |
| Throat/sputum samples |  |  |  |  |  |  |  |  |
| *Klebsiella* | 0.14 | 0.00 | 0.00 | 0.00 | 0.00 | 0.27 | 0.44 | 1.00 |
| *Haemophilus influenzae* | 0.06 | 0.00 | 0.13 | 0.00 | 0.00 | 0.00 | 0.09 | 0.33 |
| *Streptococcus* (any) | 0.83 | 0.32 | 1.02 | 0.90 | 0.47 | 0.95 | 1.31 | 0.33 |
| *Streptococcus* Group A | 0.09 | 0.00 | 0.25 | 0.10 | 0.00 | 0.14 | 0.00 | 0.00 |
| *Streptococcus* Group B | 0.12 | 0.00 | 0.13 | 0.10 | 0.19 | 0.14 | 0.18 | 0.00 |
| *Streptococcus* Group C | 0.02 | 0.00 | 0.00 | 0.10 | 0.00 | 0.00 | 0.00 | 0.00 |
| *Streptococcus* Group F | 0.12 | 0.00 | 0.13 | 0.10 | 0.09 | 0.14 | 0.26 | 0.00 |
| *Streptococcus* Group G | 0.48 | 0.32 | 0.51 | 0.50 | 0.19 | 0.54 | 0.88 | 0.33 |
