# Supplementary material for: “Epidemiology and aetiology of influenza-like illness among households in metropolitan Vientiane, Lao PDR”: A prospective, community-based cohort study
Source: PLoS One. 2019 Apr 5;14(4):e0214207. doi: 10.1371/journal.pone.0214207 (PMC6450629; doi:10.1371/journal.pone.0214207)
Supplement: S5 Table — (DOCX) [file pone.0214207.s005.docx]

**S5 Table:** Analysis of association between symptoms and pathogens

| Pathogen | Associated symptoms ^a^ | OR | Sensitivity | Specificity | PPV | NPV | *P* |
| --- | --- | --- | --- | --- | --- | --- | --- |
| FluA (any) | Fever (measured) | 1.91 (1.10,3.31) | 0.41 (0.29,0.54) | 0.73 (0.69,0.77) | 0.16 (0.11,0.23) | 0.91 (0.87,0.93) | 0.028 |
|  | Sorethroat | 2.47 (1.22,5.00) | 0.84 (0.72,0.92) | 0.33 (0.28,0.37) | 0.14 (0.10,0.17) | 0.94 (0.89,0.97) | 0.015 |
|  | Myalgia | 3.07 (1.52,6.21) | 0.84 (0.72,0.92) | 0.38 (0.33,0.42) | 0.15 (0.11,0.19) | 0.95 (0.90,0.97) | 0.002 |
| FluA/H3N2 | Sorethroat | 2.78 (1.28,6.03) | 0.85 (0.73,0.93) | 0.33 (0.28,0.37) | 0.12 (0.09,0.16) | 0.95 (0.91,0.98) | 0.011 |
|  | Myalgia | 2.97 (1.42,6.22) | 0.83 (0.71,0.92) | 0.37 (0.33,0.42) | 0.13 (0.10,0.17) | 0.95 (0.91,0.98) | 0.004 |
| FluB | Fever (measured) | 3.79 (2.02,7.12) | 0.57 (0.41,0.72) | 0.74 (0.70,0.78) | 0.16 (0.11,0.23) | 0.95 (0.93,0.97) | 0.000 |
| Rhino | Headache | 2.54 (1.21,5.36) | 0.82 (0.68,0.91) | 0.36 (0.32,0.41) | 0.11 (0.08,0.15) | 0.95 (0.91,0.98) | 0.018 |
| Cor (any) | Myalgia | 1.90 (1.07,3.38) | 0.76 (0.65,0.86) | 0.37 (0.33,0.42) | 0.16 (0.12,0.20) | 0.91 (0.86,0.95) | 0.038 |
| Cor_229 | Cough (-) | 0.26 (0.09,0.75) | 0.71 (0.44,0.90) | 0.10 (0.07,0.12) | 0.02 (0.01,0.04) | 0.91 (0.80,0.97) | 0.025 |
| Cor_63 | Dyspnea (-) | 3.51 (1.30,9.51) | 0.67 (0.41,0.87) | 0.64 (0.59,0.68) | 0.06 (0.03,0.10) | 0.98 (0.96,0.99) | 0.018 |
|  | Myalgia | 9.66 (1.27,73.1) | 0.94 (0.73,1.00) | 0.36 (0.32,0.41) | 0.05 (0.03,0.08) | 0.99 (0.97,1.00) | 0.015 |
| Cor_43 | Fever (any) (-) | 0.37 (0.15,0.89) | 0.82 (0.67,0.93) | 0.07 (0.05,0.10) | 0.07 (0.05,0.09) | 0.84 (0.70,0.93) | 0.047 |
|  | Fever (self-reported) (-) | 0.33 (0.14,0.77) | 0.80 (0.64,0.91) | 0.08 (0.06,0.10) | 0.06 (0.04,0.09) | 0.83 (0.69,0.92) | 0.017 |
| Para1 | Myalgia (-) | 0.23 (0.06,0.88) | 0.30 (0.07,0.65) | 0.35 (0.30,0.39) | 0.01 (0.00,0.02) | 0.96 (0.93,0.98) | 0.046 |
| Saur | Headache (-) | 0.51 (0.30,0.87) | 0.51 (0.38,0.64) | 0.33 (0.29,0.37) | 0.09 (0.06,0.12) | 0.84 (0.78,0.89) | 0.018 |
|  | Myalgia (-) | 0.54 (0.31,0.93) | 0.52 (0.38,0.65) | 0.34 (0.29,0.38) | 0.09 (0.06,0.12) | 0.85 (0.79,0.89) | 0.034 |
| HIB | Sorethroat (-) | 0.34 (0.13,0.88) | 0.44 (0.22,0.69) | 0.30 (0.26,0.34) | 0.02 (0.01,0.04) | 0.94 (0.89,0.97) | 0.040 |
|  | Dyspnea (-) | 0.09 (0.01,0.71) | 0.06 (0.00,0.27) | 0.62 (0.57,0.66) | 0.00 (0.00,0.03) | 0.95 (0.92,0.97) | 0.010 |
|  | Myalgia (-) | 0.33 (0.13,0.87) | 0.39 (0.17,0.64) | 0.34 (0.30,0.39) | 0.02 (0.01,0.04) | 0.94 (0.90,0.97) | 0.037 |
| Spneu | Fever (measured) | 2.29 (1.44,3.64) | 0.43 (0.33,0.54) | 0.75 (0.71,0.79) | 0.26 (0.19,0.34) | 0.87 (0.83,0.90) | 0.001 |
|  | Headache (-) | 0.56 (0.36,0.89) | 0.54 (0.43,0.65) | 0.33 (0.28,0.37) | 0.14 (0.10,0.18) | 0.78 (0.72,0.84) | 0.020 |
|  | Myalgia (-) | 0.30 (0.19,0.49) | 0.41 (0.30,0.52) | 0.31 (0.26,0.35) | 0.10 (0.07,0.14) | 0.73 (0.66,0.79) | <0.001 |
|  | Cyanosis | 7.94 (1.31,48.2) | 0.03 (0.01,0.10) | 1.00 (0.98,1.00) | 0.60 (0.15,0.95) | 0.84 (0.81,0.87) | 0.040 |
| Hinfl (culture) | Chills | 19.71 (1.75,221) | 0.67 (0.09,0.99) | 0.91 (0.88,0.93) | 0.04 (0.00,0.14) | 1.00 (0.99,1.00) | 0.017 |
| Strep_G (culture) | Fever (any) (-) | 0.29 (0.11,0.75) | 0.78 (0.58,0.91) | 0.08 (0.05,0.10) | 0.04 (0.03,0.07) | 0.86 (0.73,0.95) | 0.019 |
|  | Fever (self-reported) (-) | 0.31 (0.12,0.81) | 0.78 (0.58,0.91) | 0.08 (0.06,0.11) | 0.04 (0.03,0.07) | 0.87 (0.74,0.95) | 0.031 |
