# Supplementary material for: “Epidemiology and aetiology of influenza-like illness among households in metropolitan Vientiane, Lao PDR”: A prospective, community-based cohort study
Source: PLoS One. 2019 Apr 5;14(4):e0214207. doi: 10.1371/journal.pone.0214207 (PMC6450629; doi:10.1371/journal.pone.0214207)
Supplement: S6 Table — (DOCX) [file pone.0214207.s006.docx]

**S6 Table**: Frequency of antimicrobial resistance among bacterial cultures from throat and sputum specimens

|  |  | No. (%) of cultures | | | | | | | | | |
| --- | --- | --- | --- | --- | --- | --- | --- | --- | --- | --- | --- |
| Antibiotic | AMR test result | Streptococcus | | | | | *Haemophilus influenzae* | *Klebsiella pneumoniae* | *K. pneumoniae* and *S. aureus* | *E. coli* | Total |
|  |  | Group A | Group B | Group C | Group F | Group G |  |  |  |  |  |
| Chloramphenicol | Resistant | 1 (25) | 0 (0) | 0 (0) | 0 (0) | 0 (0) | 1 (33.3) | - | 0 (0) | 0 (0) | 2 (3.8) |
|  | Sensitive | 3 (75) | 7 (100) | 1 (100) | 7 (100) | 28 (100) | 2 (66.7) | - | 1 (100) | 1 (100) | 50 (96.2) |
| Tetracycline | Intermediate | 0 (0) | 0 (0) | 0 (0) | 0 (0) | 3 (10.7) | 0 (0) | - | 0 (0) | 0 (0) | 3 (5.8) |
|  | Resistant | 3 (75) | 7 (100) | 0 (0) | 3 (42.9) | 21 (75) | 1 (33.3) | - | 0 (0) | 1 (100) | 36 (69.2) |
|  | Sensitive | 1 (25) | 0 (0) | 1 (100) | 4 (57.14) | 4 (14.29) | 2 (66.67) |  | 1 (100) | 0 (0) | 13 (25) |
| Ofloxacin | Resistant | 0 (0) | 0 (0) | 0 (0) | 0 (0) | 0 (0) | - | - | - | - | 0 (0) |
|  | Sensitive | 4 (100) | 7 (100) | 1 (100) | 7 (100) | 28 (100) | - | - | - | - | 47 (100) |
| Ampicillin | Resistant | - | - | - | - | - | 2 (66.7) | - | - | 1 (100) | 11 (84.6) |
|  | Sensitive | - | - | - | - | - | 1 (33.3) | - | - | 0 (0) | 2 (15.4) |
| Augmentin | Resistant | - | - | - | - | - | 0 (0) | 1 (11.1) | - | 0 (0) | 1 (9.1) |
|  | Sensitive | - | - | - | - | - | 1 (100) | 8 (88.9) | - | 1 (100) | 10 (90.9) |
| Ceftriaxone | Resistant | - | - | - | - | - | 0 (0) | 1 (11.1) | - | 1 (100) | 2 (15.4) |
|  | Sensitive | - | - | - | - | - | 3 (100) | 8 (88.9) | - | 0 (0) | 11 (84.6) |
| Cotrimoxazole | Resistant | - | - | - | - | - | 3 (100) | 1 (11.1) | - | 1 (100) | 5 (38.5) |
|  | Sensitive | - | - | - | - | - | 0 (0) | 8 (88.9) | - | 0 (0) | 8 (61.5) |
| Ciprofloxacin | Intermediate | - | - | - | - | - | 0 (0) | 1 (12.5) | - | 0 (0) | 1 (8.3) |
|  | Resistant | - | - | - | - | - | 0 (0) | 0 (0) | - | 1 (100) | 1 (8.3) |
|  | Sensitive | - | - | - | - | - | 3 (100) | 7 (87.5) | - | 0 (0) | 10 (83.3) |
