# Supplementary figures and images for: “Epidemiology and aetiology of influenza-like illness among households in metropolitan Vientiane, Lao PDR”: A prospective, community-based cohort study
Source: PLoS One. 2019 Apr 5;14(4):e0214207. doi: 10.1371/journal.pone.0214207 (PMC6450629; doi:10.1371/journal.pone.0214207)

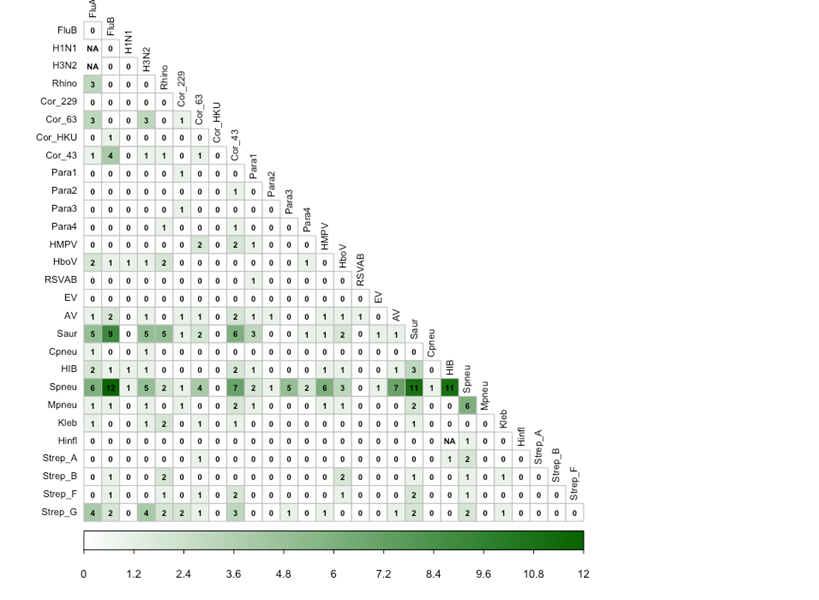

Supplement: S1 Fig — The number and color within each square represent the number of co-infections involving each pairwise combination of pathogens. (TIF) [file pone.0214207.s007.tif]

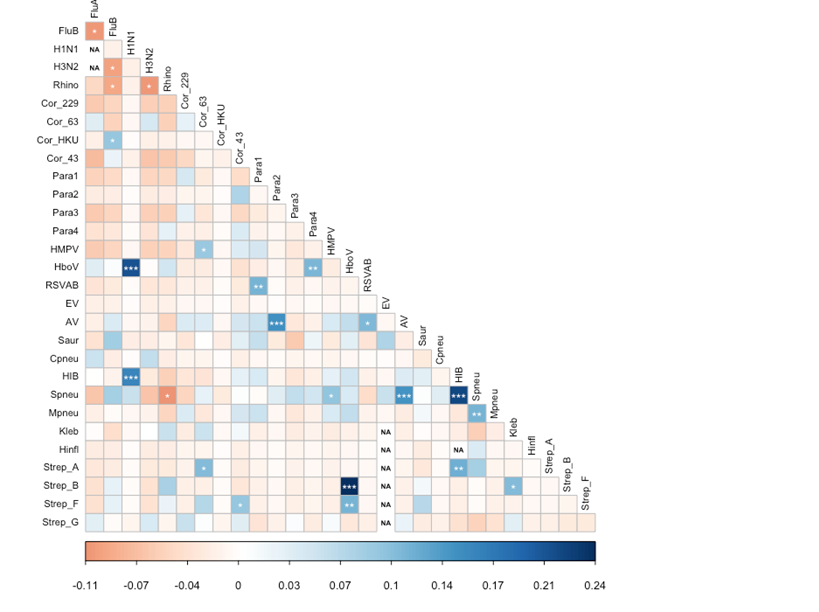

Supplement: S2 Fig — The color within each square represents the Spearman correlation coefficient (red is negative, blue is positive). Symbols *, **, and *** indicated statistically significant correlation at P<0.05, <0.01, and <0.001, respectively. Correlation coefficients could not be calculated between enterovirus (EV) and cultured pathogens (Klebsiella pneumonia, Haemophilus influenzae and Streptococcus), as microbial culture tests were not conducted for the single EV positive case. (TIF) [file pone.0214207.s008.tif]
